# Melioidosis Vaccines: A Systematic Review and Appraisal of the Potential to Exploit Biodefense Vaccines for Public Health Purposes

**DOI:** 10.1371/journal.pntd.0001488

**Published:** 2012-01-31

**Authors:** Sharon J. Peacock, Direk Limmathurotsakul, Yoel Lubell, Gavin C. K. W. Koh, Lisa J. White, Nicholas P. J. Day, Richard W. Titball

**Affiliations:** 1 Department of Microbiology and Immunology, Faculty of Tropical Medicine, Mahidol University, Bangkok, Thailand; 2 Mahidol-Oxford Tropical Medicine Research Unit, Faculty of Tropical Medicine, Mahidol University, Bangkok, Thailand; 3 Department of Medicine, Cambridge University, Addenbrooke's Hospital, Cambridge, United Kingdom; 4 Department of Tropical Hygiene, Faculty of Tropical Medicine, Mahidol University, Bangkok, Thailand; 5 Nuffield Department of Clinical Medicine, Centre for Clinical Vaccinology and Tropical Medicine, University of Oxford, Churchill Hospital, Oxford, United Kingdom; 6 College of Life and Environmental Sciences, University of Exeter, Exeter, United Kingdom; Charles Darwin University, Australia

## Abstract

**Background:**

*Burkholderia pseudomallei* is a Category B select agent and the cause of melioidosis. Research funding for vaccine development has largely considered protection within the biothreat context, but the resulting vaccines could be applicable to populations who are at risk of naturally acquired melioidosis. Here, we discuss target populations for vaccination, consider the cost-benefit of different vaccination strategies and review potential vaccine candidates.

**Methods and Findings:**

Melioidosis is highly endemic in Thailand and northern Australia, where a biodefense vaccine might be adopted for public health purposes. A cost-effectiveness analysis model was developed, which showed that a vaccine could be a cost-effective intervention in Thailand, particularly if used in high-risk populations such as diabetics. Cost-effectiveness was observed in a model in which only partial immunity was assumed. The review systematically summarized all melioidosis vaccine candidates and studies in animal models that had evaluated their protectiveness. Possible candidates included live attenuated, whole cell killed, sub-unit, plasmid DNA and dendritic cell vaccines. Live attenuated vaccines were not considered favorably because of possible reversion to virulence and hypothetical risk of latent infection, while the other candidates need further development and evaluation. Melioidosis is acquired by skin inoculation, inhalation and ingestion, but routes of animal inoculation in most published studies to date do not reflect all of this. We found a lack of studies using diabetic models, which will be central to any evaluation of a melioidosis vaccine for natural infection since diabetes is the most important risk factor.

**Conclusion:**

Vaccines could represent one strand of a public health initiative to reduce the global incidence of melioidosis.

## Author Summary

The designation of *Burkholderia pseudomallei* as a category B select agent has resulted in considerable research funding to develop a protective vaccine. This bacterium also causes a naturally occurring disease (melioidosis), an important cause of death in many countries including Thailand and Australia. In this study, we explored whether a vaccine could be used to provide protection from melioidosis. An economic evaluation based on its use in Thailand indicated that a vaccine could be a cost-effective intervention if used in high-risk populations such as diabetics and those with chronic kidney or lung disease. A literature search of vaccine studies in animal models identified the current candidates, but noted that models failed to take account of the common routes of infection in natural melioidosis and major risk factors for infection, primarily diabetes. This review highlights important areas for future research if biodefence-driven vaccines are to play a role in reducing the global incidence of melioidosis.

## Introduction

*Burkholderia pseudomallei* has been the subject of intensive research over the past decade following its classification by the CDC as a category B select agent [1]. The potential for this bacterium to cause clinical disease (melioidosis) after inhalation, coupled with the low infective dose by this route and the ease with which the bacterium can be obtained and cultured are characteristics of a pathogen that might be used for malevolent purposes. Much recent research on *B. pseudomallei* has focused on identifying ways in which the bacterium causes disease with a view to devising biodefense vaccines, and significant progress has been made in demonstrating the feasibility of immunization against melioidosis in animal models [2].

Unlike many of the other biothreat organisms, *B. pseudomallei* is also an important cause of naturally acquired human infection. This organism is present in the environment across much of SE Asia and N Australia, and infection results from bacterial inoculation, inhalation or ingestion. Most cases are reported from NE Thailand and N Australia, although melioidosis has been increasingly reported in the Indian subcontinent, China, the Middle East, Africa and South America [3], [4]. In NE Thailand, melioidosis is the third most common cause of death from infectious diseases, exceeded only by HIV and tuberculosis [5]. In Darwin, N Australia, melioidosis is the commonest cause of fatal community-acquired septicemic pneumonia [6]. *B. pseudomallei* is inherently resistant to many antibiotics, including first, second and third generation cephalosporins, aminoglycosides, penicillins and polymyxin [7], making the treatment of melioidosis difficult. The associated mortality rate is 43% in NE Thailand [5] and 14% in N Australia [8]. Against this background, there may be an opportunity to use vaccines devised for biodefense purposes for the control of naturally acquired infection. Here, we discuss target populations for vaccination, consider the cost-benefit of different vaccination strategies, and review potential vaccine candidates.

## Methods

### Cost-effectiveness analysis of vaccination against melioidosis

**Figure 1:**
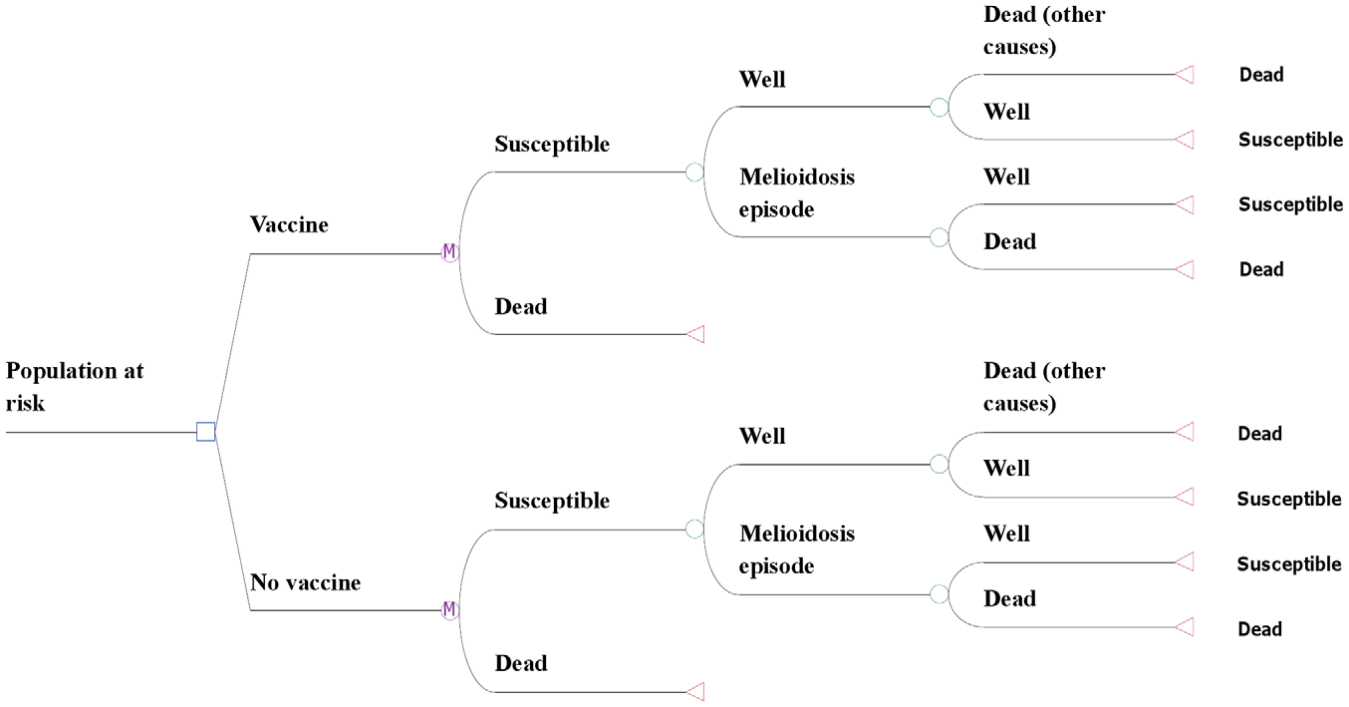
The Markov model used to assess costs and health gains for a melioidosis vaccine. *M* denotes a Markov node where individuals can transition into the subsequent states in each monthly cycle. In each cycle a susceptible patient can be infected and develop a melioidosis episode, from which they can recover and return to the susceptible state in the next cycle, or die. Patients can also die from natural causes according to their age specific mortality rates.

We considered the cost-effectiveness of vaccination against melioidosis in NE Thailand, though our models could be applied to other areas of the world where the disease is endemic. We used a Markov model to estimate cost and quality adjusted life years (QALYs) as patients transition between different health states (Figure 1). Table 1 lists the values used for the variables in the model. Our analysis considered the potential benefits of a melioidosis vaccine to reduce (i) disease incidence, and (ii) mortality with varying degrees of protective efficacy for each of these actions. The protective efficacy (PE) and protective duration (PD) of the vaccine were assumed to be homogenous for all routes of disease acquisition. Health benefits were assessed in terms of QALYs gained [9]. The incremental cost-effectiveness ratio (ICER) for a QALY gained was compared with the GDP per capita, which is a proxy measure for the assumed willingness to pay for a QALY gained [10], [11]. We assumed that Thailand was willing to pay (WTP) $3,000 for an additional QALY gained which approximates the Thai GDP/capita [10]. Given the uncertainties surrounding the PE and PD of a potential vaccine together with its costs and disease incidence in different target populations, results are presented for a broad but plausible range of estimates for each of these parameters. The cost-effectiveness analysis was carried out using Treeage Pro (TreeAge Software Inc., Williamstown, MA, USA).

**Table 1:** Data used in the cost-effectiveness model.

Inputs	Value	Sources
Incidence of melioidosis in NE Thailand		[5]
- In the general population	21.0 per 100,000 person yrs	
- In people older than 35 years old	26.2 per 100,000 person yrs	
- In people with diabetes	145.7 per 100,000 person yrs	
Mortality rate of melioidosis in NE Thailand	43%	[5]
Population of NE Thailand	21.4 million	[5]
Willingness to pay for a quality-adjusted life year (QALY) gained in Thailand	$3,000	[10]

Parameters	Range of parameters
Vaccine efficacy	
- Reduction in incidence of melioiodosis	0 to 100% reduction
- Reduction in mortality from melioidosis	0 to 100% reduction
Protective duration	1 to 15 years
Cost of vaccine	$1 to $50

### Search strategy and selection criteria

We performed a PubMed (MEDLINE) search of the literature using the keywords “*pseudomallei*”, “vaccine”, “immunity” and “protect”, and reviewed the available references published between January 1911 and Oct 2011. The inclusion criterion used was publication of the vaccine efficacy in humans or in animal models of melioidosis. Initially, titles and abstracts were screened. Articles identified as possibly relevant were reviewed as full text. The reference lists of included articles were assessed for further relevant publications. To inform discussion on the development of vaccine candidates, the animal models used for the vaccine trials were also reviewed.

## Results and Discussion

### Target population for a melioidosis vaccine

Countries that might consider using a biodefense vaccine for the control of endemic melioidosis include Thailand and Australia, where the most reliable estimates of the incidence of melioidosis are available [4]. The annual incidence of human melioidosis in NE Thailand and the Top End of N Australia are currently 21.0 and 19.6 per 100,000 population, respectively [5], [12]. However, incidence rates are not uniform within the population, with certain populations at particularly high risk, including those with diabetes mellitus, chronic lung disease or chronic kidney failure [5], [12]. For example, the annual incidence rates in diabetics have been estimated to be 145.7 and 260.4 per 100,000 population in NE Thailand and N Australia, respectively [5], [12]. The annual incidence rates in patients in N Australia with chronic lung disease or chronic kidney disease have been estimated to be 102.0 and 119.6 per 100,000 population, respectively [12]. People older than 35 years are also at higher risk (26.2 versus 4.3 per 100,000 population per year for over or under 35 years of age, respectively) [5].

High-risk groups could be considered as primary targets for melioidosis vaccine trials. One disadvantage may be that generating protective immunity in individuals with such underlying diseases may be difficult to achieve. Furthermore, limiting vaccination to people with diabetes, chronic lung disease or chronic kidney failure would only capture around 60% to 70% of all melioidosis cases [5], [12]. In addition, around 15% of patients presenting with melioidosis have previously undiagnosed diabetes. An alternative target group would be all people over 35 years of age residing in an area where melioidosis is known to occur. Vaccinating this much larger group would be predicted to be less cost-effective, but would capture the majority of possible melioidosis patients.

### Routes of *B. pseudomallei* infection and implications for vaccine development

The commonest routes of *B. pseudomallei* infection are thought to be inoculation, inhalation and ingestion [13]. The prevailing assumption is that most naturally occurring disease results from percutaneous inoculation [13]. This is largely based on the observation that people at high risk such as agricultural workers do not wear protective clothing, work with bare feet, and suffer repeated minor injuries. In addition, disease incidence increases during the rainy season when rice farmers have regular and prolonged contact with contaminated soil and water [8], [14]. Although entirely feasible, this association is not supported by published evidence. A retrospective study performed in N Australia found that less than one quarter of people presenting with melioidosis recalled an injury in the preceding weeks [6], and a case-control study conducted in the same setting found that exposure to soil was not associated with melioidosis [15]. Inhalation of *B. pseudomallei* suspended in aerosols generated from the environment was considered to be an important mechanism for infection in US combatants during the conflict with Vietnam, particularly in helicopter crewmen [16]. Published evidence for inhalation as a route of infection in the general population is limited to several studies from N Australia that reported a shift towards a higher frequency of pneumonia and severe disease during the rainy season or following heavy monsoon rains and winds [17], [18]. There is also evidence for ingestion as an important route of *B. pseudomallei* infection. Several clusters of melioidosis cases have been reported from Australia in which a strain of *B. pseudomallei* isolated from a common water source was a genetic match for the strain causing disease in the cluster [19], [20]. The probability of this occurring by chance is small since *B. pseudomallei* is genetically extremely diverse [21]. *B. pseudomallei* has also been isolated from public water supplies in 11 locations in the Northern Territory of Australia, genotyped and implicated as a source of infection in 6 locations [22]. In addition, acute suppurative parotitis, which is common in pediatric melioidosis patients in NE Thailand, is presumed to result from direct entry of organisms present in the mouth. In the absence of information on the relative importance of each route of infection it is clear that a melioidosis vaccine for public health purposes should protect against oral, inhalational and percutaneous challenges.

### Cost-effectiveness of vaccination against melioidosis

The model used was based on the effect of a full course of vaccine, which could be either a single inoculation or multiple inoculations. Two target populations were considered: (i) all individuals older than 35 years of age; or (ii) a high-risk group with diabetes mellitus, chronic lung disease or chronic kidney failure. We considered situations where protective efficacy (PE) of a vaccine course ranged from 0 to 100%, protective duration (PD) ranged from 1 to 15 years, and the cost of a full vaccine course ranged from $1 to $50 (Table 1). PE was assumed to be homogenous across the target population.

**Figure 2:**
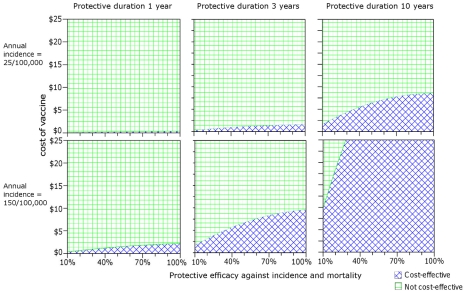
Cost-effectiveness of melioidosis vaccines as determined by incidence, cost, protective efficacy and protective duration. It was assumed that policy makers in Thailand were willing to pay $3,000 for an additional QALY gained. Areas in blue indicate where the vaccine is considered cost-effective in the Thai context. The protective efficacy (PE) and protective duration (PD) of the vaccine were considered as homogenous for all routes of disease acquisition. Percentage reduction of PE in this figure was considered as a combination of reduction in both disease incidence and mortality rate.

Figure 2 shows some situations where a vaccine would be predicted to be cost-effective. With a PD of one year, a vaccine was only cost-effective in the group with major risk factors (estimated annual incidence of 150 per 100,000 persons) and provided that the PE was over 50% and cost was less than $2. If the PD was 3 years, a vaccine at the same price and PE would be cost effective in all adults with an average incidence of 25 per 100,000. If the PD was 3 years, PE was 100%, and only high risk group were targeted, the full vaccine course could rise to $10, and still represent a cost-effective intervention. A vaccine course that reduced both incidence rate and mortality of melioidosis by 50% with a PD of at least 10 years could cost up to $10 in the general adult population and over $ 25 in diabetics and maintain its cost-effectiveness.

The potential number of deaths averted and costs associated with different vaccine target populations in NE Thailand assuming that the PE of a vaccine was 50% are shown in Table 2. The estimates are for a single cycle, and so both the costs and potential number of deaths averted would repeat themselves at the end of the duration of PE.

**Table 2:** Cost and deaths averted if a melioidosis vaccine^a^ was implemented in NE Thailand.

Target group	Population in NE Thailand	Protective duration	Deaths prevented	Cost (single cycle)
people older than 35 years old	∼9.5 million	1 year	612	$48 million
		3 years	1838	
		10 years	6127	
people with diabetes	∼250,000	1 year	121	$1.3 million
		3 years	363	
		10 years	1209	

a The melioidosis vaccine was assumed to have 50% protective efficacy (reduction of disease incidence by 50% and reduction of mortality rate in diseased patients by 50%) and cost 5 dollars.

### Vaccine candidates

**Figure 3:**
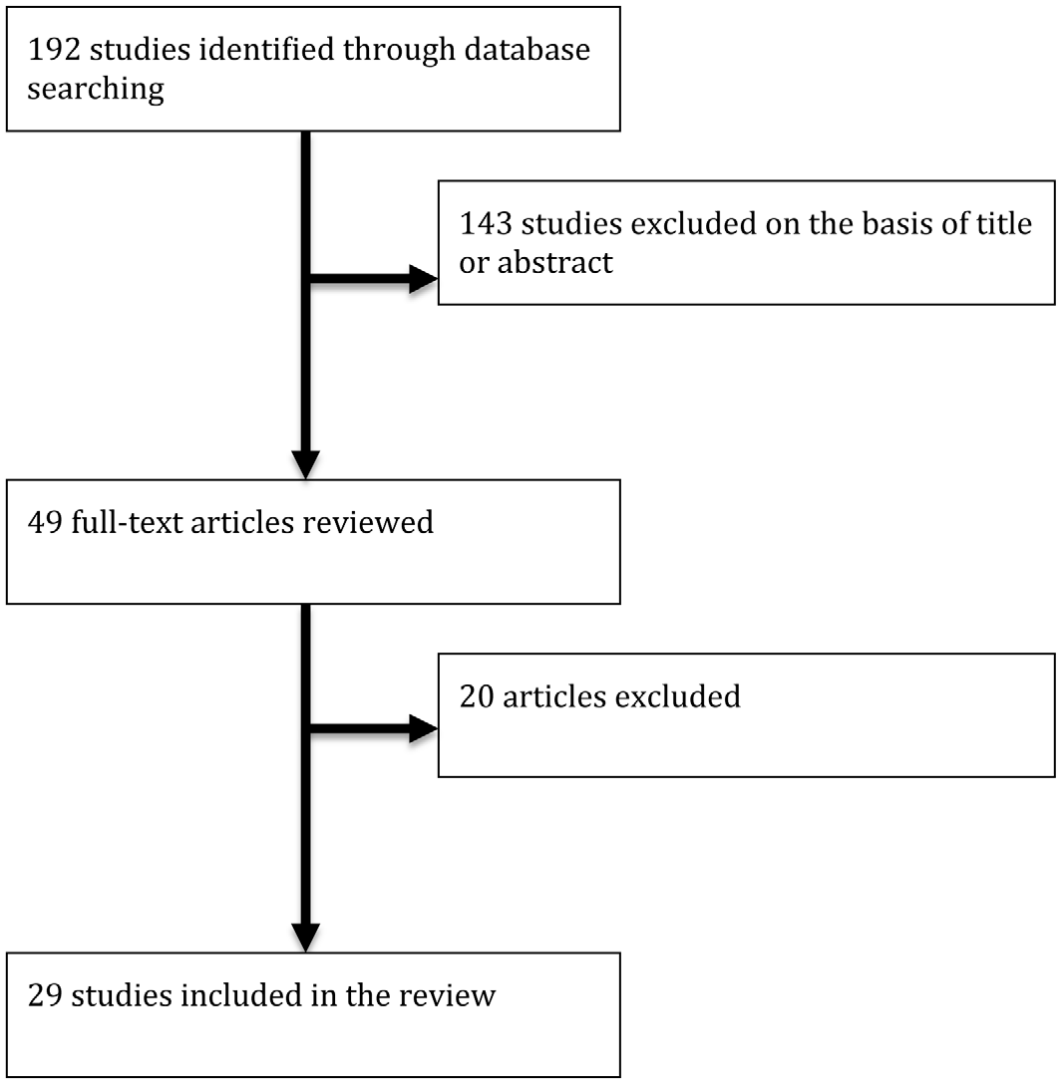
Study flow diagram.

We identified 29 studies that examined the following vaccine types: live attenuated (n = 11), whole cell killed (n = 5), subunit (n = 9), plasmid DNA (n = 2) and dendritic cell (n = 2) (Figure 3). All of the vaccine candidates were evaluated in mouse models, but the *B. pseudomallei* strains used, doses and routes of lethal challenge were highly variable (Table S1). Sterile immunity was rarely reported. Vaccines being developed for biodefence purposes would need to protect primarily against an inhalation challenge. The available experimental evidence indicated that this might be challenging, since the protective efficacy of tested vaccines was greater against intraperitoneal challenge compared with inhalation or intranasal challenge (Table S1). Studies of protection following an ingestion challenge have not been reported.

### Live attenuated vaccines

A wide range of attenuated *B. pseudomallei* mutants have been reported, and immunization of mice with some of these has resulted in the induction of protective immunity [23], [24], [25], [26], [27], [28], [29], [30], [31], . Live attenuated mutants of *B. pseudomallei* have been shown to be capable of inducing protection against either an injected or an intranasal challenge, but protection was strongly dependent on immunization by the same route as challenge (Table S1). A potential advantage of a live attenuated vaccine is the likely ability to induce long-term protection against disease [34]. For example, the live attenuated tularemia vaccine induces cell-mediated responses which persist for at least 3 decades [35] and immunity after vaccination with vaccinia virus persists for decades [36] and possibly for the lifetime of the individual [37]. A live attenuated vaccine against melioidosis that induced long-term protection is likely to be highly cost-effective. However, it may prove difficult to license a live attenuated mutant for use in humans in endemic areas. This bacterium has the potential to cause a potentially life-threatening disease that is difficult to treat, and one would need confidence that reversion of an attenuated mutant to virulence was not possible. In addition, there is a concern that an attenuated mutant might become established as a latent infection. This is found on observation that *B. pseudomallei* can survive for extend periods in the human host, the longest reported duration of naturally acquired latency prior to clinical symptoms being 62 years [38]. Concerns over the use of a live attenuated meliodosis vaccine are heightened by the knowledge that most naturally occurring cases of disease occur in individuals who are likely to have some degree of immune dysfunction.

### Inactivated whole cell vaccines

Inactivated vaccines are used widely to protect against viral infections, but there are few examples of inactivated bacterial vaccines in current use. Inactivated vaccines are relatively easy and cheap to produce and are capable of inducing protective immunity that persists for several years. For example, protective immunity after immunization with inactivated *Salmonella enterica* serovar Typhi is reported to persist for at least 30 months [39], [40]. A potential advantage of killed cell vaccines is their ability to present a wide range of antigens to the immune system. This might be important when considering the genetic and immunological diversity of *B. pseudomallei*. Kill *B. pseudomallei* has resulted in the induction of protective immunity [41], [42], [43]. Killed *B. thailandensis*, a closely related but avirulent organism, was able to induce comparable protection against killed *B. pseudomallei* [44], and intranasal inoculation of killed *B. pseudomallei* plus adjuvant CLDC (cationic lipid-DNA complex) gave protection from lethal pulmonary challenge [45]. The main disadvantage of killed cell vaccines is the potential for short-term but undesirable side effects. In other killed cell vaccines, these side effects are largely attributed to the pyrogenic effects of the lipid A portion of lipopolysaccharide (LPS) [46]. However, *B. pseudomallei* LPS is reported to be at least ten times less potent in eliciting nitric oxide and tumor necrosis factor α from macrophages than LPS from *Escherichia coli* or *Salmonella enterica* [47], [48]. In addition, *B. pseudomallei* LPS is less potent than enterobacterial LPS in the induction of pyrogenic activity in rabbits and lethality in galactosamine-sensitized mice [49]. Thus some of the concerns over the use of killed cell vaccines for melioidosis may not be justified, although detailed studies with a killed cell vaccine would be required to confirm the safety and lack of reactogenicity of such a preparation in animal models and humans.

### Sub-unit vaccines

Sub-unit vaccines incorporate specific molecules derived from a microorganism and are the aspiration of most vaccine research programmes. The potential advantages of these vaccines lie with their increased safety and ability to evoke immune responses only to the protective antigen rather than to the entire microorganism. The duration of protection elicited after immunization with a sub-unit vaccine may differ markedly from vaccine to vaccine and between different population. For example, polysaccharide vaccines often elicit short-lived responses, especially in infants [50]. However, many protein-based vaccines, such as tetanus toxoid and hepatitis B vaccine, can elicit protective immunity which persists for at least a decade [51], [52].

A range of proteins and polysaccharides have been identified that induce different degrees of protection against an intraperitoneal *B. pseudomallei* challenge in mice [53], [54], [55], [56], [57], [58], [59], [60], [61]. Of these, the most protective appear to be LPS, capsular polysaccharide (CPS), LolC protein (an inner membrane protein which forms part of a lipoprotein export system), an outer membrane protein Omp85, and Hcp2 (integral surface-associated component of T6SS). A minority of *B. pseudomallei* strains produce LPS with an immunologically distinct O-antigen [62], raising the possibility that LPS may not induce protective immunity against all strains. There are no reports of the ability of these individual sub-units to induce significant protection against an inhalation challenge, which would be essential for a biodefense vaccine. However, immunization of mice with outer membrane vesicles, which are likely to contain a combination of sub-units, can provide protection against a low-level (5 LD_50_ doses) inhalation challenge [61]. There is accumulating evidence, therefore, that subunit vaccines devised for biodefence use may be suitable for use in populations in melioidosis endemic areas.

### Naked DNA and dendritic cell vaccines

There are two reports on the protection afforded by immunisation with DNA vaccines encoding the *B. pseudomallei* flagellar subunit gene, *fli*C [63], [64]. These showed that immunization of mice with these constructs provided modest levels of protection. However, in general naked DNA vaccines against infectious diseases have been weakly immunogenic in humans even though they have promoted vigorous and effective immune responses in mice [65]. To overcome these limitations prime-boost strategies have often been adopted to allow the development of protective immunity [65]. Two studies have been published on dendritic cells pulsed with heat-killed whole cell *B. pseudomallei* [66], [67]. Dendritic cell vaccines have been increasingly evaluated in clinical trials for cancer [68], and there is still a need to undertake more proof of principle studies evaluating their utility as vaccines for infectious diseases [69].

### Animal models

Suitable animal models of infection will be central to any melioidosis vaccine development programme. A myriad of different infection models are available, reflecting the different forms of disease in humans. Acute disease occurs in some mouse strains (e.g. BALB/c), and in hamsters with death typically occurring within 7 days of challenge by the intraperitoneal route. Challenge of BALB/c mice by the inhalation or intranasal route results in hyperacute disease, with death within a few days. C57BL/6 mice are typically 10^4^-fold more resistant to disease than BALB/c mice [31], and are more likely to develop a chronic form of the disease which shows similarities with chronic melioidosis in humans [70]. Chronic melioidosis can also be observed in BALB/c mice after low-dose exposure [71], [72]. There are two reports of disease after enteral challenge of mice with *B. pseudomallei* [73], but there is a clear need for the further development of animal models of disease following ingestion of the bacteria.

Diabetic mouse models of melioidosis have not been well described [74], [75], but are highly desirable for evaluating vaccines for natural infection. Mice can be made diabetic for extended periods of time by dosing with streptozotocin, and dendritic cells and macrophages isolated from streptozotocin-induced diabetic mice have altered abilities to ingest and kill *B. pseudomallei* [76]. Young diabetic rats were found to be susceptible to *B. pseudomallei*, but not adult diabetic rats [74], [75], which is not comparable to the natural situation in humans in which diabetics with melioidosis are predominantly adults.

Efficacy studies in non-human primates are also likely to be required for approval of melioidosis vaccine in human clinical trials. Although mouse models may be useful to screen vaccine candidates, the patterns of disease and immune responses are often different from those seen in human. A range of different non-human primate infection models including marmoset and macaque are currently being developed, but there are currently no reported data on the suitability of these models for testing vaccine candidates.

The form of the disease may also be dependent on the infecting strain. *B. pseudomallei* is a genetically diverse genus, and there are significant differences in the virulence of different strains of *B. pseudomallei*, at least in BALB/c mice. There is some evidence of genetic variation in strains from different continents, suggesting that it will be important to test vaccines with strains typical of those encountered by the target population. The finding that some strains produce an atypical and immunologically distinct O-antigen means that it may be desirable to test these strains in efficacy studies. However, the low frequency with which naturally occurring disease caused by these strains is encountered also indicates that such testing could be relatively limited. It is of undoubted importance, however, to identify a panel of *B. pseudomallei* isolates that are representative of the genetic diversity of the bacterium in the range of countries in which the vaccine will be used. Vaccine efficacy should then be evaluated using this test panel with the same standard dosages and multiple routes of inoculation.

### Concluding comments

We have demonstrated that within a plausible range of estimates for cost and efficacy, it is possible that a vaccine would be cost-effective for the prevention of naturally acquired melioidosis. In Thailand, a vaccine is more likely to be a cost-effective intervention if used in high-risk populations such as diabetics and possibly in other at-risk groups such as those working in rice fields. In countries with a higher GDP, it might be cost effective to immunize the entire population in endemic areas as well as tourists or military personnel deploying to melioidosis endemic areas. The finding that a range of candidate vaccines achieved partial protection against disease in mice suggests potential for human use. Reduction in disease severity alone would be predicted to improve outcome in view of the high mortality rate and the fact that half of all in-patient deaths in Thailand occur within the first 48 hours as a result of septic shock. Additional research is required to investigate the potential synergies between vaccination, early antimicrobial treatment and improved care of sepsis in resource-restricted settings. Further studies are also required to determine whether immunization with the best vaccine candidates protects against multiple routes of disease acquisition, is effective in diabetic patients, and not accompanied by unacceptable side effects.

## Supporting Information

Table S1 **Studies of melioidosis vaccines in animal models.**
(DOC)

Checklist S1 **PRISMA checklist.**
(DOC)
