# Correction: Melioidosis Vaccines: A Systematic Review and Appraisal of the Potential to Exploit Biodefense Vaccines for Public Health Purposes

**DOI:** 10.1371/annotation/20664ec9-17e4-4ba6-8c65-481a70a5c4ad

**Published:** 2013-02-26

**Authors:** Sharon J. Peacock, Direk Limmathurotsakul, Yoel Lubell, Gavin C. K. W. Koh, Lisa J. White, Nicholas P. J. Day, Richard W. Titball

Table S1 contains two items that require correction. The OMV vaccine given subcutaneously is listed as providing 0% protection against challenge; in fact, it provided 60% protection. Additionally, the challenge information should indicate 1000 CFU by aerosol instead of 5000 CFU intranasally.

Please view the correct Table S1 here: 
