# Supplementary material for: Melioidosis Vaccines: A Systematic Review and Appraisal of the Potential to Exploit Biodefense Vaccines for Public Health Purposes
Source: PLoS Negl Trop Dis. 2012 Jan 31;6(1):e1488. doi: 10.1371/journal.pntd.0001488 (PMC3269417; doi:10.1371/journal.pntd.0001488)
Supplement: Table S1 — Studies of melioidosis vaccines in animal models. (DOC) [file pntd.0001488.s001.doc]

Table S1. Studies of melioidosis vaccines in animal models

| Ref. | Vaccine | Animal model | Immunization route* | | Challenge in BALB/c mice model | | | % survival |
| --- | --- | --- | --- | --- | --- | --- | --- | --- |
|  |  |  |  |  | Strain | Route* | Dose  (CFU) |  |
| ***Live attenuated vaccines*** | | | | | | | | |
|  | Acapsular polysaccharide mutant | BALB/c and Porton mice | i.p. | | 576 | i.v. | 1 x 10^4^ | 0% at d 18 |
|  | *ilvI* (branched chain amino acid synthesis) mutant | BALB/c mice | i.p. | | 576 | i.p. | 1 x 10^6^ | 80% at d 35 |
|  | *ilvI* (branched chain amino acid synthesis) mutant | BALB/c mice | i.p. | | 576 | i.p. | 1 x 10^6^ | 40% at d 75 |
|  | *BipD* (type III secretion system) mutant | BALB/c mice | i.p. | | 576 | i.p. | 1 x 10^4^ | 60% at d 72 |
|  | *serC* (Serine biosynthesis) mutant | BALB/c mice | i.p. | | 576, K96243 | i.p. | 1 x 10^4^ | >70% at d 30 |
|  | *aroB* (Aromatic amino acid biosynthesis) mutant | BALB/c mice | i.n. | | K96243 | i.n. | 1 x 10^3^ | 0% at d 10 |
|  | *purN* (Purine biosynthesis) mutant | BALB/c mice | i.p. | | E8 | i.v. | 1 x 10^3^ | 0% at d 35 |
|  | *purN* (Purine biosynthesis) mutant | BALB/c mice | i.n. | | E8 | i.n. | 1 x 10^3^ | 0% at d 35 |
|  | *aroC* (Sucrose biosynthesis) mutant | BALB/c & C57BL/6 mice | i.p. | | A2 | i.p. | 6 x 10^3^ | 80% at 5 mo |
|  | CL04 strain from a patient with chronic melioidosis | BALB/c & C57BL/6 mice | Unknown | | NTCC 13178 | Unknown | 7 x 10^2^ | 73% at d 18 |
|  | *asd* (aspartate-β-semialdehyde dehydrogenase) mutant | BALB/c | i.n. | | 1026b | i.n. | 4 x 10^3^ | 20% at d 40 |
|  | *ilvI* mutant with CpG oligodeoxynucleotide | BALB/c mice | i.p. | | 576 | i.n. | 1 x 10^2^ | 0% at d 15 |
|  | *ilvI* mutant with CpG oligodeoxynucleotide | BALB/c mice | i.n. | | 576 | i.n. | 1 x 10^2^ | 40% at d 15 |
| ***Killed whole cell vaccines*** | | | | | | | | |
|  | Heat killed *B. pseudomallei*, unknown strain | Unknown | | i.p. | Unknown | i.p. | 2 x 10^6^ | 100% at d 7 |
|  | Heat killed *B. pseudomallei* strain NCTC13179 | BALB/c mice | | s.c. | NCTC13179 | i.v. | 20 | 0% at d 40 |
|  | Heat killed LPS-negative *B. pseuodmallei* mutant | BALB/c mice | | i.p. | K96243 | i.p. | 6.5 x 10^4^ | 80% at d 35 |
|  | Heat killed *B. pseudomallei* strain 576 | BALB/c mice | | i.p. | K96243 | i.p. | 4 x 10^4^ | 50% at d 45 |
|  | Heat killed *B. pseudomallei* strain K96243 | BALB/c mice | | i.p. | K96243 | i.p. | 4 x 10^4^ | 50% at d 45 |
|  | Heat killed *B. pseudomallei* strain K96243 | BALB/c mice | | i.p. | K96243 | i.n. | 92 | 0% at d 45 |
|  | Heat killed *B. mallei* strain 23344 | BALB/c mice | | i.p. | K96243 | i.p. | 4 x 10^4^ | 70% at d 45 |
|  | Heat killed *B. thailandensis* strain E27 | BALB/c mice | | i.p. | K96243 | i.p. | 4 x 10^4^ | 60% at d 45 |
|  | Heat killed *B. pseudomallei* 1026b with CLDC adjuvants | BALB/c mice | | i.n. | 1026b | i.n. | 7.5 x 10^3^ | 44% at d 40 |
| ***Subunit vaccines*** | | | | | | | | |
|  | Capsular polysaccharide | BALB/c mice | | i.p. | NCTC4845 | i.p. | 2 x 10^4^ | 0% at d 28 |
|  | Lipopolysaccharide | BALB/c mice | | i.p. | NCTC4845 | i.p. | 2 x 10^4^ | 50% at d 35 |
|  | Capsular polysaccharide | BALB/c mice | | i.p. | NCTC4845 | i.n. | 12.5 | 0% at d 5 |
|  | Lipopolysaccharide | BALB/c mice | | i.p. | NCTC4845 | i.n. | 12.5 | 0% at d 5 |
|  | *LolC* (ATP binding cassette system) | BALB/c mice | | i.p. | K96243 | i.p. | 4 x 10^4^ | 80% at d 42 |
|  | *LolC* (ATP binding cassette system) | BALB/c mice | | i.p. | 576 | i.p. | 6.6 x 10^5^ | 30% at d 42 |
|  | Bip proteins | BALB/c mice | | i.p. | Ashdown | i.p. | 970 | 0% at d 5 |
|  | Omp3 and Omp7 (Outer membrane proteins) | BALB/c mice | | i.p. | D286 | i.p. | 1 x 10^6^ | 50% at d 21 |
|  | Omp85 (Outer membrane protein) | BALB/c mice | | i.p. | D286 | i.p. | 1 x 10^6^ | 70% at d 15 |
|  | Peptide mimotopes of EPS | BALB/c mice | | i.v. | NCTC4845 | i.p. | 4.7 x 10^4^ | 0% at d 30 |
|  | *Hcp2* (intergral surface-associated component of T6SS) | Syrian hamster | | i.p. | K96243 | i.p. | 5 x 10^5^ | 83% at d 42 |
|  | Lipopolysaccharide from *B. thailandensis* | BALB/c mice | | i.p. | K96243 | i.p. | 2 x 10^4^ | 50% at d 35 |
|  | Outer membrane vesicle | BALB/c mice | | s.c. | 1026b | i.n. | 5 x 10^3^ | 0% at d 14 |
|  | Outer membrane vesicle | BALB/c mice | | i.n. | 1026b | i.n. | 5 x 10^3^ | 20% at d 14 |
| ***Naked DNA vaccines*** | | | | | | | | |
|  | *fli*C gene | BALB/c mice | | i.m. | 16 local strains | i.v. | 1 x 10^5^ | 83% at d 7 |
|  | *fli*C gene with CpG oligodeoxynucleotide | BALB/c mice | | i.m. | 16 local strains | i.v. | 1 x 10^5^ | 93% at d 14 |
| ***Dendritic cell DNA vaccines*** | | | | | | | | |
|  | Dendritic cell pulsed with heat-killed whole cell *B. pseudomallei* | BALB/c mice | | i.d. and i.n. | NCTC4845 | i.p. | 5.3 x 10^4^ | 60% at d 35 |
|  | Dendritic cell pulsed with heat-killed whole cell *B. pseudomallei* in combination with CpG ODN | BALB/c mice | | i.d. | NCTC4845, K96243, 576 | i.p. | 1 x 10^4^ | 77% at d 42 |

* i.p. (intraperitoneal), i.v. (intravenous), i.n. (intranasal), i.m. (intramuscular), s.c. (subcutaneous)
